# An Active Absorbent for Cleanup of High-Concentration Strong Acid and Base Solutions

**DOI:** 10.3390/ma12203389

**Published:** 2019-10-17

**Authors:** Nara Han, Sol Park, Byung Kwon Kaang, Wooree Jang, Hye Young Koo, Won San Choi

**Affiliations:** 1 Department of Chemical and Biological Engineering, Hanbat National University, 125 Dongseodaero, Yuseong-gu, Daejeon 305-719, Korea; xoc123@naver.com (N.H.); thf6363@naver.com (S.P.); rkdqudrnjs3@naver.com (B.K.K.); 2 Functional Composite Materials Research Center, Korea Institute of Science and Technology (KIST) Jeonbuk Institute of Advanced Composite Materials, 92 Chudong-ro, Bongdong-eup, Wanju-gun, Jeollabuk-do 55324, Korea; 215015@kist.re.kr

**Keywords:** chemical absorbents, iron oxide, strong acid and base, drop test, dust test

## Abstract

There is significant interest in developing novel absorbents for hazardous material cleanup. Iron oxide-coated melamine formaldehyde sponge (MFS/IO) absorbents with various IO layer thicknesses were synthesized. Various other absorbents were also synthesized and compared to evaluate the absorption capability of the MFS/IO absorbents for strong acid (15%, v/v) and base (50%, m/m) solutions. Specifically, absorbent and solution drop tests, dust tests, and droplet fragment tests were performed. Among the various absorbents, MFS/IO absorbents possessing a needlelike surface morphology showed several unique characteristics not observed in other absorbents. The MFS/IO absorbents naturally absorbed a strong base solution (absorption time: 0.71–0.5 s, absorption capacity: 10,000–34,000%) without an additional external force and immediately absorbed a strong acid solution (0.31–0.43 s, 9830–10,810%) without absorption delay/overflow during absorbent and solution drop tests, respectively. The MFS/IO absorbents were also demonstrated to be ideal absorbents that generated fewer dust particles (semiclass 1 (ISO 3) level of 280 piece/L) than the level of a clean room (class 100). Furthermore, the MFS/IO absorbents were able to prevent the formation of droplet fragments and solution overflow during the solution drop test due to their unique surface morphology and extremely high absorption speed/capacity, respectively.

## 1. Introduction

Hazardous materials, including strong acids/bases, oils, and solvents, are widely used in manufacturing industries in general [1,2,3,4]. Spillages of these hazardous materials have devastating consequences for both human health and the environment. Thus, industries have made efforts to appropriately treat hazardous materials after use. Many approaches for hazardous material cleanup have been proposed, including neutralization, dilution, washing, bioremediation, solidification, or absorption [5,6,7,8,9,10]. Among these methods, the absorption of hazardous materials using absorbents is a convenient and efficient strategy for cleanup of hazardous materials, and there is significant interest in developing novel absorbents to remove hazardous materials. However, most previous studies have focused on synthesizing hydrophobic absorbents to remove oils and organic solvents [11,12,13,14,15,16,17,18,19,20,21,22,23]. Relatively little attention has been paid to hydrophilic absorbents that can remove aqueous hazardous materials [24,25,26]. In particular, there are only a limited number of reports on the development of acid/base absorbents [27]. Absorbents for removing strong acid and base solutions are commercially available, but these absorbents do not meet the desired criteria for strong acid and base absorbents and have very low absorption efficiencies. To the best of our knowledge, an active absorbent that meets the requirements for cleanup of high-concentration strong acid and base solutions and can be employed in a clean room has not yet been reported.

Strong acids and bases are used for cutting, etching, and cleaning Si wafers during semiconductor manufacturing processes [28,29,30]. Strong acid/base waste solutions and residues should be properly removed during the processes. Chemical absorbent foams and pads have been used to remove the strong acid/base waste solutions produced during these processes. For a large amount of solution, an absorbent foam is dropped onto the solution to naturally absorb the solution [5,6]. For a small amount of solution, absorbent pads are normally placed below the conveyor belt or process system to absorb the generated residue or waste solution [5,6]. However, previously reported or produced absorbent foams and pads have difficulty spontaneously absorbing strong acid/base solutions, i.e., without an external force, immediately absorbing solutions without an absorption delay, and efficiently avoiding the formation of droplet fragments. Furthermore, various additional tests have not yet been conducted for absorbent applications in special fields. The currently available absorbents do not have the necessary properties to be used for the absorption of high-concentration strong acid/base solutions. A good absorbent must satisfy certain criteria: i) Once an absorbent is in contact with a solution, the absorbent should naturally and automatically absorb the solution without the use of an additional external force because the manufacturing process utilizing strong acid/base, i.e., semiconductors, prohibits human involvement; for example, etching processes are normally performed by unmanned systems due to the emission of strong acids/bases; ii) the solution should be immediately absorbed without an absorption delay to prevent overflow when a solution is added to an absorbent; iii) the concentration of dust particles generated during the use of the absorbent should be extremely low because products, i.e., semiconductors, are manufactured in clean rooms; and iv) the formation of droplet fragments should be avoided or minimized to prevent secondary contamination when a solution is dropped on an absorbent.

Herein, we report an MFS/IO absorbent possessing a needlelike surface morphology. This MFS/IO absorbent naturally absorbs a strong base solution (50%) without an additional external force and immediately absorbs a strong acid solution (15%) without absorption delay/overflow during absorbent and solution drop tests, respectively. This MFS/IO absorbent is also demonstrated to be an ideal absorbent that generates fewer dust particles than the level of a clean room (class 100) and prevents the formation of droplet fragments during solution drop tests. 

## 2. Materials and Methods 

### 2.1. Materials

Iron (II) sulfate heptahydrate (FeSO_4_·7H_2_O, 99.0%), iron (III) sulfate hydrate (Fe_2_(SO_4_)_3_·xH_2_O, 97%), oxalic acid (98%), tin (II) chloride (98%) and sodium hydroxide were purchased from Sigma-Aldrich. Hydrochloric acid (>35%) was purchased from Daejung Chemicals (St. Louis, MO, USA). The melamine formaldehyde sponge (MFS) and cotton were purchased from BASF and Hanmi Towel, respectively. A commercial chemical absorbent was purchased from Schoeller (St. Gallen, Switzerland). All chemicals were used without further purification. Deionized (DI) water with a resistance of 18.2 MΩ cm^−1^ was obtained from a Millipore Simplicity 185 system.

### 2.2. Preparation of MFS/IO(1–5)

A mixture of iron(II) sulfate heptahydrate (18.5 mg), iron(III) sulfate hydrate (34.8 mg), and DI water (10 mL) was prepared and stirred at room temperature for 1 min. Three milliliters of the abovementioned mixture was added to fresh DI water (10 mL). A piece of MFS (2 × 2 × 2 cm^3^) was immersed in the resulting solution at room temperature for 4 h with vigorous shaking. The resulting MFS, MFS/IO(1), was gently washed three times with DI water and dried at room temperature. The experiment was repeated until the desired number of IO layers was obtained to prepare MFS/IO(1–5).

### 2.3. Preparation of MFS/SnO_2_(1–3)

Oxalic acid (0.5 g) was added to DI water (50 mL) and stirred at room temperature for 15 min. As soon as pristine MFS (2 × 2 × 2 cm^3^) was immersed in the resulting solution, tin chloride (1 g) was added to the solution, which was then stirred at room temperature for 1 h. The resulting MFS, MFS/SnO_2_(1), was washed with DI water and dried at 80 °C overnight. The experiment was repeated until the desired number of SnO_2_ layers was obtained to prepare MFS/SnO_2_(1–3).

### 2.4. Absorbent or Solution Drop Tests

For the absorbent drop test, various absorbents (1 × 1 × 1 cm^3^) were dropped into strong acid (15% HCl) and base (50% NaOH) solutions from a height of 5 cm above the surface of the solutions. For the solution drop tests, various absorbents (2 × 2 × 1 cm^3^) were placed on a table, and 5 mL of a strong acid solution (15% HCl) was dropped from a height of 3 cm above the surface of the absorbents. All tests were performed based on the standard for hazardous materials treatment of Samsung Electronics [31]. Recyclability tests of MFS/IO(1–5) and MFS/SnO2(1–3) for the 1st to 3rd uses for the absorption of a NaOH or HCl solution were performed by absorption/squeezing/washing/drying processes. After squeezing, each absorbent was washed 3 times with DI water and dried at 80 °C for 4 h.

### 2.5. Dust Test (Static and Dynamic Mode Tests)

A test box (45 × 30 × 30 cm^3^) was placed in a clean room (class 1000, ISO 6). All dust tests were conducted in a clean room to measure the concentration of dust particles generated by each absorbent. For the static mode test, droplets of DI water (8 mL) were dropped for 10 min from a height of 30 cm above the surface of each absorbent (4 × 4 × 1 cm^3^). The number of dust particles generated by each absorbent was measured by a particle counter every minute. For the dynamic mode test, each absorbent (2 × 2 × 1 cm^3^) was attached to the bar of a metronome. The beat of the metronome was set to 0.67~1 Hz. The number of dust particles generated by each absorbent was measured by a particle counter every minute.

### 2.6. Characterization 

Scanning electron microscopy (SEM) analyses were performed using a Hitachi S-4800 instrument (20 keV, Tokyo, Japan). MFS/IO(1–5) sample was cut into a small piece (5 × 5 × 5 mm^3^) for SEM measurement. X-ray diffraction (XRD) patterns were obtained on a Rigaku X-ray diffractometer (Billerica, MA, USA) equipped with a Cu Kα source. TGA was performed using a thermogravimetric analyzer (Sinco TGA N-1500, Waltham, MA, USA) over a temperature range of 25–800 °C at a heating rate of 10 °C/min under air (flow rate, 60 cm^3^/min). Particle counter analyses were performed using the XINTEST HT-9601 instrument (Guangdong, China). The contact angle measurements were carried out using a contact angle meter (SEO Phoenix 300Touch, Gilbert, AZ, USA) at ambient temperature, and the volume of the probing liquid was 20 μL.

## 3. Results and Discussion

### 3.1. Characterization of MFS/IO 

**Figure 1:**
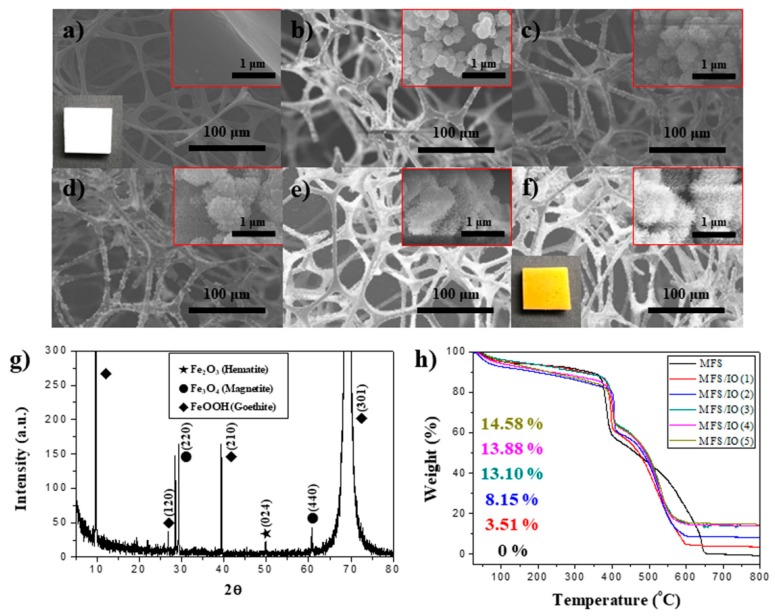
SEM images of MFS (**a**) before and (**b**–**f**) after IO synthesis. (**a**) MFS, (**b**) MFS/IO(1), (**c**) MFS/IO(2), (**d**) MFS/IO(3), (**e**) MFS/IO(4), and (**f**) MFS/IO(5). (**g**) XRD data of MFS/IO(3) as a representative of the IO-coated MFS samples. (**h**) TGA data for MFS and MFS/IO(1–5). Insets of (**a**) and (**f**) show the images of MFS and MFS/IO(5), respectively.

Figure 1 shows scanning electron microscopy (SEM) images of the iron oxide-coated melamine formaldehyde sponge (MFS/IO) in each cycle. The MFS/IO (n = 1–5) with various IO layer thicknesses was prepared by varying the number of IO coatings. MFS had an interconnected 3D-network skeleton and a smooth surface morphology, whereas the surface morphology of MFS/IO(1) consisted of 275-nm-sized IO particles with a bumpy surface structure on the MFS surface (Figure 1a,b). As the number of IO layers increased [MF/IO(2–5)], the bumpy structure on the particles gradually changed into a jagged surface morphology, and the overall size of the IO particles increased to 1 µm because of the growth of needlelike particles (Figure 1c–f). The needle-like surface morphology of MFS/IO(1–5) can possess a significant number of air pockets within the structure. The MFS/IO color changed from white to yellow-red (insets of Figure 1a,f). No detached fragments were observed upon handling MFS/IO(1–5). The IO species were identified via X-ray diffraction (XRD) measurements. The XRD pattern showed three IO peaks corresponding to hematite, magnetite, and goethite, demonstrating the successful synthesis of IO on MFS (Figure 1g). The TGA data revealed that MFS/IO(1–5) possessed IO content of 3.51–14.58%, confirming that the IO content of MFS/IO can be tuned (Figure 1h). 

### 3.2. Drop Tests of Various Absorbents with a Strong Base Solution 

To investigate the effect of the dissolution and surface morphology of the absorbent on the absorption capability of the absorbent, various absorbents with different surface chemistries and morphologies were tested. To test the absorption capability of IO-coated MFS (MFS/IO) for a strong base solution (50%), various potential candidate samples were prepared, tested, and compared. Namely, an absorbent drop test was performed [5,6,31]. The absorption properties of each absorbent were monitored after a piece of absorbent (1 × 1 × 1 cm^3^) was gently dropped on a strong base solution from a height of 5 cm. 

**Figure 2:**
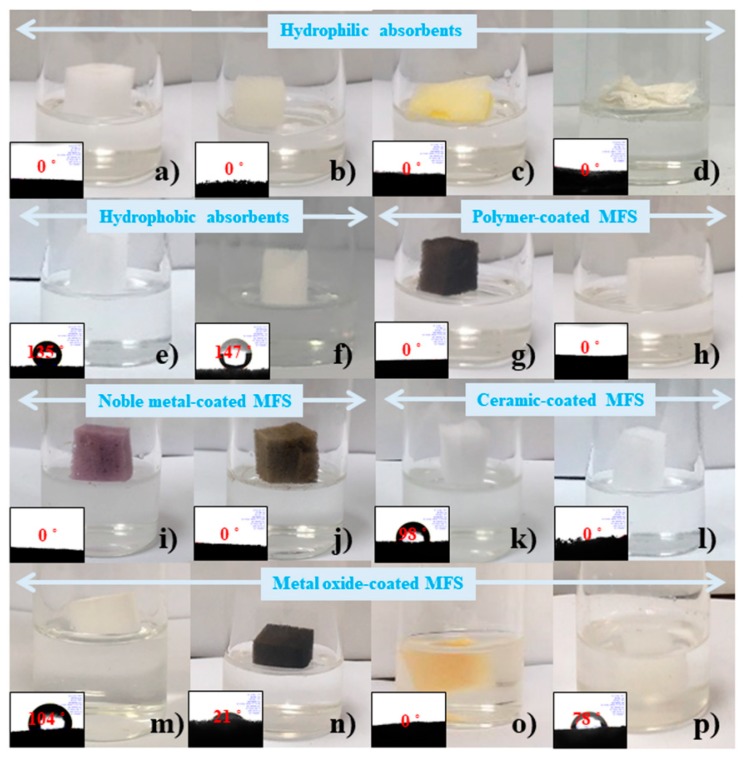
Drop tests of various absorbents with a strong base solution (50% NaOH). (**a**–**d**) Superhydrophilic absorbents. (**a**) MFS, (**b**) PUS, (**c**) absorption pad, and (**d**) cotton piece. (**e**,**f**) Hydrophobic material-coated MFS samples: (**e**) PDMS- and (**f**) ODA-coated MFS. (**g**,**h**) Superhydrophilic polymer-coated MFS samples: (**g**) Pdop- and (**h**) polyelectrolyte brush-coated MFS. (**i**,**j**) Noble metal-coated MFS samples: (**i**) Au- and (**j**) Ag-coated MFS. (**k**,**l**) Ceramic-coated MFS samples: (**k**) SiO_2_- and (**l**) CaCO_3_-coated MFS. (**m**–**p**) Metal oxide-coated MFS samples: (**m**) TiO_2_-, (**n**) Fe_3_O_4_-, (**o**) iron oxide-, and (**p**) SnO_2_-coated MFS.

The superhydrophilic samples, including pristine MFS, polyurethane sponge (PUS), a commercial absorption pad (Schoeller), and cotton, floated on the surface of the solution and did not sink to the bottom. However, these samples were expected to sink because of their superhydrophilicity (Figure 2a–d). Figure 2c shows that the commercial absorption pad used to absorb dripping acid/base solutions was not suitable for absorbing the strong base solution in the absorbent drop test. As expected, the hydrophobic material (polydimethylsiloxane and octadecylamine)-coated MFS samples [24] did not absorb the strong base solution, and they did not sink (Figure 2e,f). However, even the polydopamine- and polyelectrolyte brush-coated MFS samples [32,33], which can contain large amounts of water [34], floated without absorbing the solution (Figure 2g,h). Noble metal- and ceramic-coated MFS samples were also tested. Although Au- and Ag-coated MFS [35] possess superhydrophilic characteristics, these samples did not absorb the solution (Figure 2i,j). The SiO_2_- and CaCO_3_-coated MFS [22] showed the same results (Figure 2k,l). All samples floated on the base solution and did not absorb the solution even after 3 days. Several metal oxide-coated MFS samples exhibited interesting results. The TiO_2_- and Fe_3_O_4_-coated MFS samples [24] showed phenomena analogous with the abovementioned results (Figure 2m,n). However, interestingly, the IO- and SnO_2_-coated MFS samples quickly absorbed the solution within several seconds and were submerged in the base solution (Figure 2o,p). Although the MFS/IO(1) was the lightest one in all tested cases, it quickly absorbed the base solution and was submerged in the solution, which suggests that the weight of the sponge was not the main reason for absorption of strong base solution (Figure S1).

### 3.3. Drop and Reusability Tests of MFS/IO(1–5)

**Figure 3:**
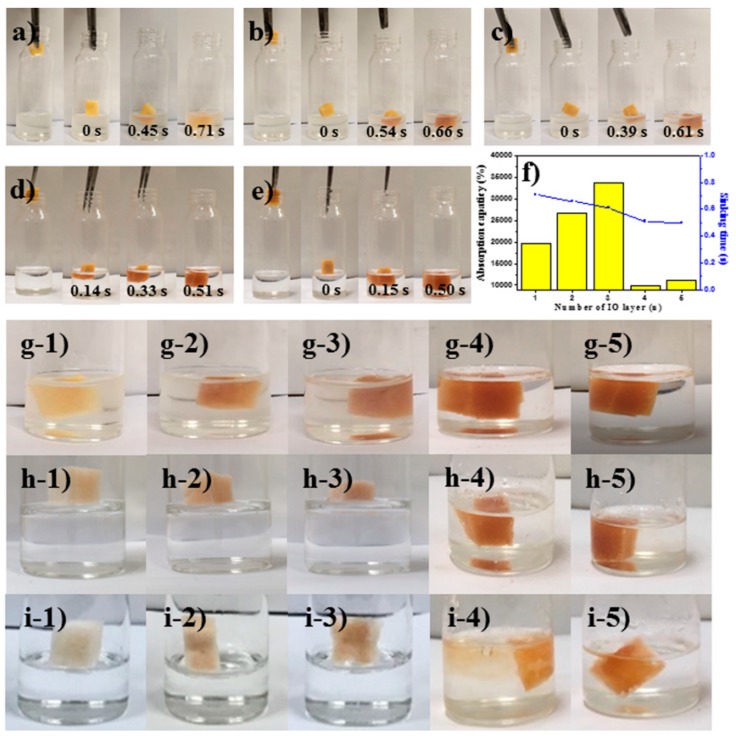
Drop tests of IO-coated MFS with a strong base solution (50% NaOH). (**a**) MFS/IO(1), (**b**) MFS/IO(2), (**c**) MFS/IO(3), (**d**) MFS/IO(4), and (**e**) MFS/IO(5). (**f**) Absorption capacity and sinking time of MFS/IO(1–5). (**g**–**i**) Reusability test of MFS/IO(1–5). Drop tests of MFS/IO(1–5) for (**g**) 1st, (**h**) 2nd, and (i) 3rd use. (**g**-**1**,**h**-**1**,**i**-**1**) MF/IO(1), (**g**-**2**,**h**-**2**,**i**-**2**) MF/IO(2), (**g**-**3**,**h**-**3**,**i**-**3**) MF/IO(3), (**g**-**4**,**h**-**4**,**i**-**4**) MF/IO(4) and (**g**-**5**,**h**-**5**,**i**-**5**) MF/IO(5).

To further investigate the absorption characteristics of MFS/IO toward a strong base solution, MFS/IO(1–5) samples with various IO layer thicknesses were tested. MFS/IO(1) rapidly absorbed the base solution and immediately sank after the piece of MFS/IO(1) was gently dropped onto the surface of the base solution (Figure 3a). In the base solution, the time required for absorption and sinking was 0.71 s for MFS/IO(1), and this time was further shortened as the thickness of the IO layer in MFS/IO(2–5) increased (Figure 3b–e). The absorption time was the same as the sinking time because the absorption time was determined by the time required for sinking. MFS/IO(5) with the thickest IO layer showed the fastest time (0.5 s) for absorbing the base solution, while MFS/IO(3) exhibited the best absorption capacity, i.e., 34,000%. To our knowledge, this absorption capacity is the highest reported absorption capacity, including oil and water. This high capacity can be ascribed to the large specific surface area of MFS/IO(3), which has chestnut burr-like particles on the MFS surface. Although MFS/IO(5) absorbed the base solution faster than MFS/IO(3), the absorption capacity of MFS/IO(5), which has a thicker IO layer than MFS/IO(3), was less than that of MFS/IO(3) (Figure 3f, yellow bar and blue line). This difference in absorption capacity can be ascribed to the larger mass of MFS/IO(5), which results in a reduced volume for absorbing the base solution. The MFS/IO samples all showed fast absorption times (0.71–0.5 s) and excellent absorption capacities (10,000–34,000%) in their 1st use (Figure 3f,g1–5). When the MFS/IO(1–5) samples were reused, MFS/IO(1), MFS/IO(2), and MFS/IO(3) did not absorb the solution and floated on the solution in the 2nd and 3rd cycles (Figure 3h1–3,i1–3). However, MFS/IO(4) and MFS/IO(5) exhibited excellent recyclability up to the 3rd cycle with high absorption capacities (1398–8076%) (Figure 3h4–5,i4–5 and Figure S2). MFS/IO(1), MFS/IO(2), and MFS/IO(3) were initially yellow-red in color but became slightly discolored after the 1st use, and they did not absorb the base solution in the 2nd and 3rd cycles (Figure 3g1–3,h1–3,i1–3). In contrast, MFS/IO(4) and MFS/IO(5) still exhibited their original color after the 1st use and continued to absorb the base solution in the 2nd and 3rd cycles (Figure 3g4–5,h4–5,i4–5). These results suggest that the absorption capability of MFS/IO for a strong base solution is related to the dissolution of IO in MFS/IO. Partial dissolution of MFS/IO was observed during the absorption of the base solution. After the 1st use, the chestnut burr-like particles on the MFS surface disappeared due to IO dissolution for MFS/IO(1) and MFS/IO(2), which resulted in relatively smooth IO surfaces (Figure 1b,c and Figure S3). For MFS/IO(4) and MFS/IO(5), although most of the chestnut burr-like particles disappeared, the densely packed needlelike IO surface morphology remained (Figure 1e,f and Figure S3). After the 1st use, MFS/IO(1) and MFS/IO(2) with smooth IO surfaces could not absorb the base solution, while MFS/IO(4) and MFS/IO(5) with needlelike IO surfaces could be further used. These results suggest that the absorption capability of MFS/IO toward a strong base solution is affected by both the dissolution and surface morphology of IO. Although the abovementioned MFS/Fe_3_O_4_ contained IO, MFS/Fe_3_O_4_ with a smooth surface did not dissolve in the strong base solution and did not absorb the solution (Figure 2n and Figure S4), which means that the surface morphology of MFS/IO mainly affects the absorption capability of MFS/IO. This hypothesis regarding the absorption mechanism will be further discussed later.

### 3.4. Drop Tests of MFS/SnO_2_(1–3) with a Strong Base Solution

**Figure 4:**
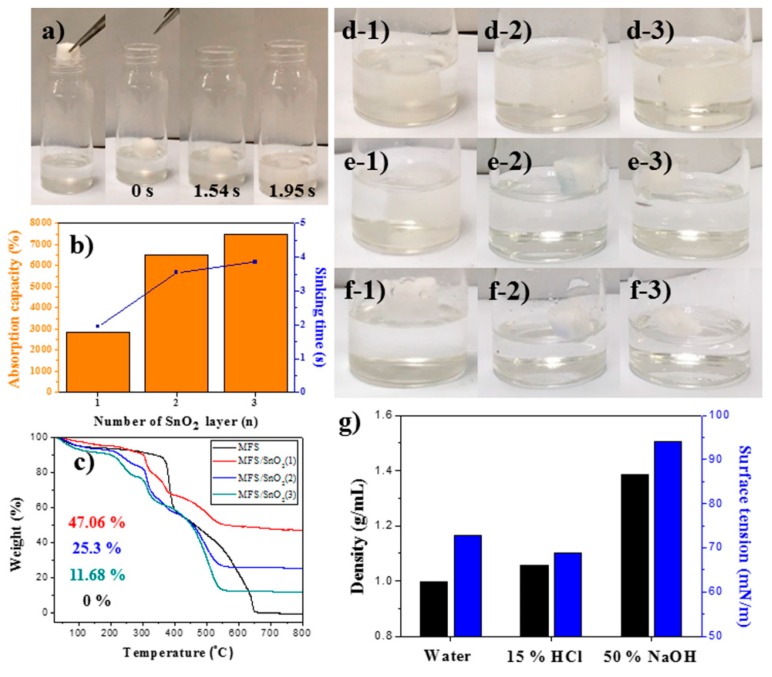
Drop tests of SnO_2_-coated MFS with a strong base solution. (**a**) MFS/ SnO_2_(1). (**b**) Absorption capacity and sinking time of MFS/SnO_2_(1–3). (**c**) TGA data of MFS/SnO_2_(1–3). (**d**–**f**) Reusability test of MFS/SnO_2_(1–3). Drop tests of MFS/SnO_2_(1–3) for (**d**) 1st, (**e**) 2nd, and (**f**) 3rd use. (**d**-**1**,**e**-**1**,**f**-**1**) MF/SnO_2_(1), (**d**-**2**,**e**-**2**,**f**-**2**) MF/SnO_2_(2), and (**d**-**3**,**e**-**3**,**f**-**3**) MF/SnO_2_(3). (**g**) Density and surface tension of strong base solution (50% NaOH).

The absorption characteristics of the MFS/SnO_2_ sample were also investigated and compared with those of the MFS/IO sample because MFS/SnO_2_ absorbed the strong base solution as well. MFS/SnO_2_(1–3) with various SnO_2_ layer thicknesses were prepared. An absorbent drop test was performed. MFS/SnO_2_(1) relatively quickly absorbed the base solution and became submerged under the solution, as observed for the MFS/IO samples (Figure 4a). The time required for absorbing and submerging under the base solution was 1.95 s for MFS/SnO_2_(1), and this time further lengthened as the SnO_2_ layer thickness increased (Figure 4b, blue line). The TGA data revealed that MFS/SnO_2_(1) and MFS/SnO_2_(3) possessed 47.06% and 11.68% and the thickest and thinnest SnO_2_ layers, respectively (Figure 4c, red and greenish blue lines). MFS/SnO_2_(1) with the thickest SnO_2_ layer exhibited the fastest time for absorbing the base solution, while MFS/SnO_2_(3), with the thinnest SnO_2_ layer, showed the best absorption capacity, reaching 7482%, which was analogous with the MFS/IO results (Figure 4b). Nevertheless, the MFS/IO samples showed a much faster absorption time and higher absorption capacity than the MFS/SnO_2_ samples for the strong base solution. The differences in density and performance of IO and SnO_2_ could be attributed to the intrinsic properties of IO and SnO_2_. MFS/SnO_2_(1) exhibited relatively good recyclability for 2 cycles, with an absorption capacity of 2,446% (Figure 4d1,e1 and Figure S2). However, MFS/SnO_2_(2) and MFS/SnO_2_(3) floated on the solution without absorption in the 2nd and 3rd cycles (Figure 4e2,f2,e3,f3). In other words, MFS/SnO_2_(2) and MFS/SnO_2_(3) with smooth SnO_2_ surfaces no longer absorbed the base solution after the 1st use, while MFS/SnO_2_(1) with a rough SnO_2_ surface continued to absorb the solution after the 1st use (Figure S5). Partial dissolution of the SnO_2_ layer of MFS/SnO_2_ was observed upon absorption of the base solution. Although the time required to absorb the base solution was slightly longer than that of MFS/IO, MFS/SnO_2_ absorbed the strong base solution only when the rough SnO_2_ surface was dissolved. The results for the MFS/IO and MFS/SnO_2_ samples indicate that a rough surface, such as a needlelike structure, is favorable for absorbing a strong base solution, which leads to dissolution of the IO and SnO_2_ layers. To confirm this hypothesis, model experiments were performed. Superhydrophilic samples with smooth and rough surfaces were prepared and used to investigate the effect of the surface morphology on the absorption of a strong base solution. 

Droplets of NaOH solution were dropped from a height of 3 cm onto each surface during the dropping tests. When a droplet of a strong base solution (50%) was dropped on a superhydrophilic Cu mesh [36] with a smooth surface, a hemispherical droplet with a water contact angle (WCA) of 91° formed, remained for 2 min, and then gradually disappeared after 10 min (Figure S6a). For the bare MFS and pad samples, the initial droplet shapes and sizes were maintained for over 2 min, and the droplets gradually disappeared after 2 min (Figure S6b,c). These results indicate that evaporation rather than absorption was the predominant influence. However, the base droplet was quickly absorbed within 0.2 s when a droplet of the strong base solution was dropped onto a superhydrophilic Cu mesh with a rough surface (Figure S6d). Furthermore, MFS/IO with a needlelike morphology exhibited the fastest absorption time (0.04–0.14 s) of all the samples (Figure S6e,f). These results confirmed our hypothesis that a rough surface is favorable for absorbing a strong base solution. A hierarchical structure, such as a needlelike morphology, can enhance mass transportation of a fluid into a hierarchical structure by capillary force, which can be observed on the surface of hierarchical structures in a fluid [37,38]. In our case, the base solution can easily infiltrate the needlelike IO structures of MFS/IO via capillary action between the base solution and needlelike structure. The capillary action will enhance the contact area between IO and the base solution, accelerate the dissolution of IO, and locally increase the concentration of iron ions (Fe^2+^/Fe^3+^) on the surface, which will induce additional flow of the base solution into the needlelike IO structures on MFS/IO to equilibrate (i.e., decrease the concentration of iron ions). Consequently, the absorption of the strong base solution by MFS/IO will be increased. To examine another reason various absorbents have difficulty absorbing strong base solutions with extremely high concentrations, the density and surface tension of the base solution were measured and compared with those of water. The density of the strong base solution (50%) was 1.384 g/mL, which is 1.4 times higher than that of water (Figure 4g, black bars). The strong base solution also showed a surface tension (93.98 mN/m) 1.3 times higher than that of water (Figure 4g, blue bars). The surface of the absorbent should first be wetted with the solution for absorption, because the high density and surface tension of the strong base solution can prevent absorbent wetting by allowing the absorbent to float on the solution. However, the MFS/IO samples decreased the abovementioned barriers and induced absorption of the solution through wetting due to the capillary action created by the needlelike morphology on MFS/IO. A lack of capillary action of absorbents and high density/surface tension of solution could be another reason various absorbents with smooth surfaces have difficulty absorbing strong base solutions.

### 3.5. Drop Test of a Strong Acid Solution

**Figure 5:**
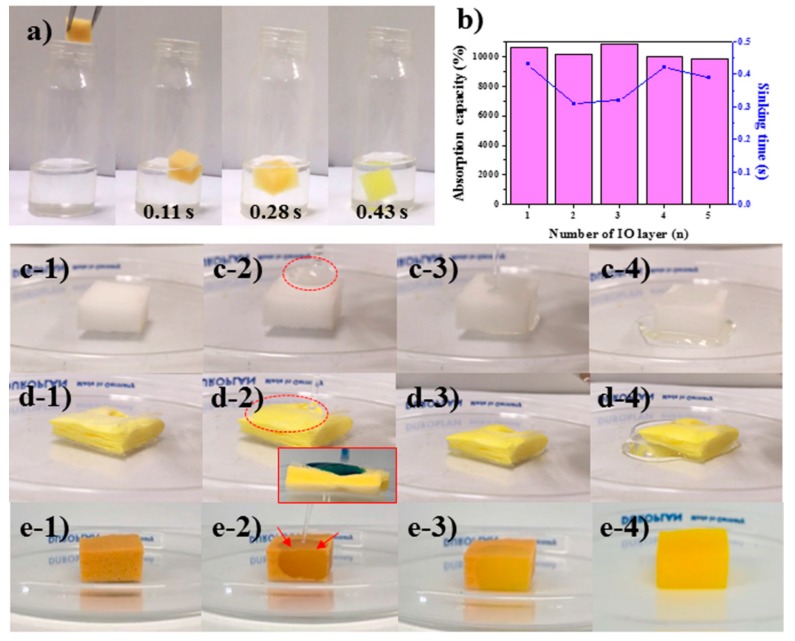
(**a**) Drop tests of MFS/IO(1) with a strong acid solution (15% HCl). (**b**) Absorption capacity and sinking time of MFS/IO(1–5) for a strong acid solution. (**c**–**e**) Solution drop tests. Drop test of a strong acid solution on (**c1**–**c4**) pristine MFS, (**d1**–**d4**) the pad, and (**e1**–**e4**) MFS/IO(3). (Inset of d2) Acidic water colored blue with methylene blue dye was used for visualization of the water stream.

To further demonstrate the prominent features of MFS/IO resulting from the needlelike IO surface morphology, a strong acid solution (15% HCl) that is widely used in semiconductor manufacturing processes was used for absorbent drop tests. Several candidate absorbents, such as pristine MFS and absorption pads, were tested for comparison. The pristine MFS and absorption pads floated on the acid solution without absorbing the solution for the 1st use (Figure S7). When a piece of MFS/IO(1) was gently dropped on the strong acid solution, MFS/IO(1) rapidly absorbed the acid solution and became submerged under the solution, as observed in the strong base solution (Figure 5a). All MFS/IO samples showed fast absorption times (0.31 s–0.43 s) and excellent absorption capacities (9,830%–10,810%) in the 1st use (Figure 5b, pink bars and blue line). As observed with the base solution, MFS/IO absorbed the acid solution during the dissolution of the IO structure. For the acid solution, MFS/IO(1–5) had analogous absorption capacities that ranged from 9,830% to 10,810%, irrespective of the IO layer thickness, which can be ascribed to complete dissolution of the needlelike IO structure on the MFS surface during the 1st use. The color of MFS/IO(1–5) was white after the 1st use, and MFS/IO(1–5) did not absorb the acid solution in the 2nd and 3rd cycles, which further indicated complete dissolution of the IO structure (Figure S8). To extend the application range, another test, a solution drop test, was performed [5,6,31]. The absorption degree and aspects of the absorbents were monitored when a strong acid solution was dropped from a certain height on the absorbents (2 × 2 × 1 cm^3^). To exclude gravity and height effects, a low drop height was selected. When a 5 mL aliquot of the acid solution with a drop velocity of 0.14 m/s was poured from a height of 3 cm onto superhydrophilic MFS, absorption of the acid solution after an absorption delay was observed (Figure 5c1–c3). Then, the acid solution overflowed from MFS (Figure 5c4), which suggested that the absorption speed and capacity of MFS were slow and insufficient, respectively, for the absorption of 5 mL of the acid solution. An absorption delay and overflow of the strong acid solution were also observed when the acid solution was poured onto a commercial absorption pad under the same conditions (Figure 5d1–d4). The overflow amount with the absorption pad was much greater than that with MFS (Figure 5c4,d4). In contrast, MFS/IO(3) quickly absorbed the acid solution without an absorption delay (Figure 5e1,e2). After pouring was complete, the acid solution was homogeneously distributed within MFS/IO(3) (Figure 5e3). No overflow was observed with MFS/IO(3) (Figure 5e4). These results indicate that MFS/IO(3) does not have an absorption delay and prevents overflow of the acid solution due to its fast absorption speed and sufficient maximum absorption capacity, respectively. In summary, MFS/IO(3) quickly absorbed the acid solution during the absorbent drop test and immediately absorbed an acid solution without absorption delay and overflow during the solution drop test. No notable reactions were observed with the naked eye during the drop tests of the solutions and absorbents.

### 3.6. Dust Tests of MFS/IO During Solution Dropping under Static and Dynamic Modes

**Figure 6:**
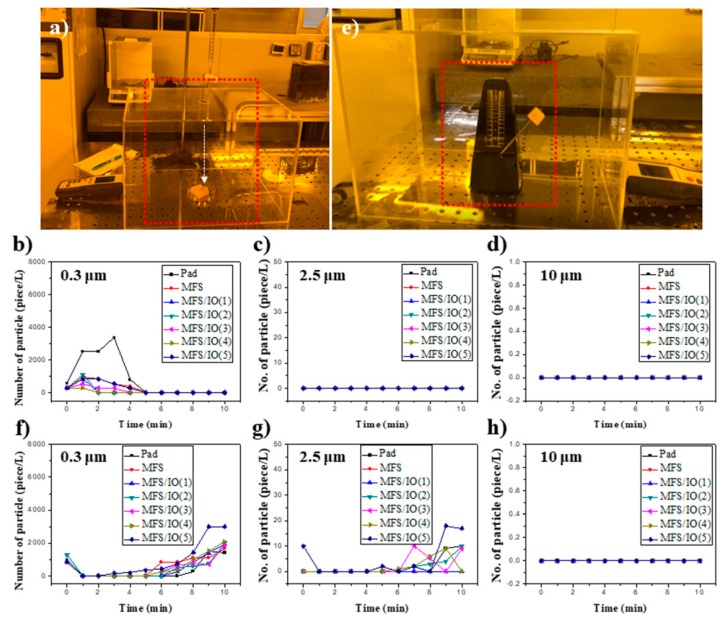
Dust tests of MFS/IO(3) during solution dropping under (**a**) static and (**e**) dynamic modes. Three types of absorbents, MFS, pad, and MFS/IO(1–5), were tested, and MFS/IO(3) was used as a representative image for the static and dynamic modes. Dust particles generated in the (**b**–**d**) static and (**f**–**h**) dynamic mode tests. The size of the dust particles generated from each absorbent: (**b**,**f**) 0.3 µm, (**c**,**g**) 2.5 µm, and (**d**,**h**) 10 µm.

To investigate the possibility of real application of the MFS/IO absorbent, two dust tests, static and dynamic, were conducted in a clean room because semiconductor manufacturing processes using strong acid/base are performed in clean rooms. Pristine MFS, a commercial absorption pad, and MFS/IO(1–5) were used for the dust tests. A test box equipped with an air particle counter to monitor dust particles and a burette to release solution was placed in the clean room (class 1000, ISO 6) (Figure 6a). For the static mode test, after placing the absorbents in the box, the concentration change in dust particles generated during the use of the absorbents was monitored. The concentration of dust particles in a semiconductor manufacturing process should be extremely low to prevent the incorporation of unwanted particles [28]. The commercial absorption pad generated a high concentration of dust particles (3361 pieces/L) with a size of 0.3 µm within 3 min when a drop of a strong acid solution was dropped onto the pad (Figure 6b, black line), indicating that a large number of dust particles detach from the commercial absorption pad during use. The concentration of dust particles generated by pristine MFS was 960 pieces/L, which was much lower than that generated by the commercial absorption pad (Figure 6b, red line). The MFS/IO(1–5) samples generated lower or analogous concentrations (1120–280 piece/L) of dust particles relative to the pad and pristine MFS (Figure 6b, blue, green, pink, mustard, and navy lines). Particularly, MFS/IO(1) and MFS/IO(3–5) showed much lower concentrations than pristine MFS, and the concentrations were at the class 10 (ISO 4) level of 280–840 piece/L. 

No additional dust particles were generated by MFS/IO(1–5) after a certain time, indicating that no additional dust particles were detached from the MFS/IO(1–5). Among the samples, MFS/IO(4) exhibited the lowest value (280 piece/L) that complies with semiclass 1 (ISO 3) level. Considering the level of a clean room (class 100–class 10,000) for semiconductor manufacturing processes, MFS/IO(1) and MFS/IO(3–5) were demonstrated to be ideal absorbents that generate dust particle concentrations below the standard level. No dust particles with sizes of over 2.5 µm were generated by pristine MFS, the commercial absorption pad, and MFS/IO(1–5) (Figure 6c,d). Although absorbents are statically used in the majority of cases, dynamic tests were also conducted for broader application and thorough verification. In the dynamic mode tests, a metronome equipped with the absorbents was used, and its speed was adjusted to 0.15 m/s (Figure 6e). Although the MFS/IO samples generated much more dust particles in the dynamic mode (1766–2090 piece/L) than the MFS/IO samples in the static mode, the dust particle concentrations were analogous to those of the pad and pristine MFS and class 100 level (Figure 6f). Although MFS/IO and the pad generated low concentrations of 2.5-µm dust particles (9–17 pieces/L), the values complied with class 100 level (Figure 6g). No dust particles with sizes over 5 µm were generated (Figure 6h). 

### 3.7. Test for the Droplet Fragments that Bounced from the Surface of Each Absorbent

**Figure 7:**
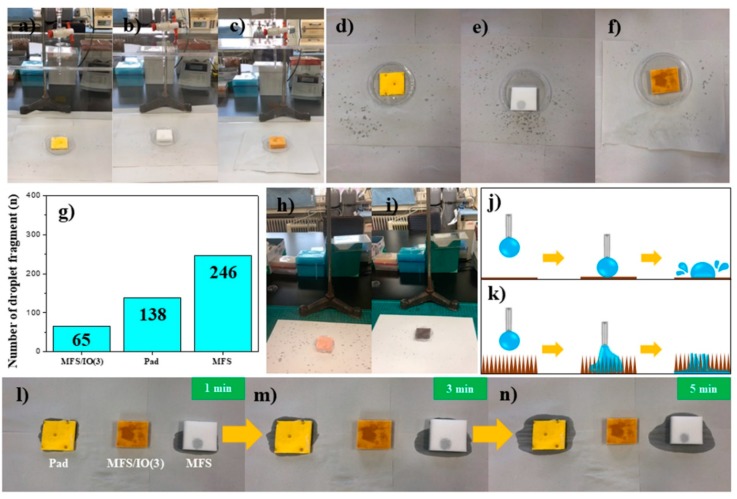
Test for the droplet fragments that bounced from the surface of each absorbent: (**a**,**d**) pad, (**b**,**e**) MFS, and (**c**,**f**) MFS/IO(3). (**g**) Number of droplet fragment particles formed from the surface of each absorbent upon acid solution dropping. Superhydrophilic Cu meshes with (**h**) smooth and (**i**) needlelike surface morphologies. Schematic representation of a plausible explanation for the droplet behaviors on (**j**) smooth and (**k**) needle-like surfaces. After dropping the acid solution for 1 min on each absorbent, the solution released from each absorbent was monitored as a function of time: (**l**) 1 min, (**m**) 3 min, and (**n**) 5 min. (left) Pad, (middle) MFS/IO(3), and (right) MFS.

To investigate the potential for real application of the MFS/IO absorbent, droplet fragment tests were also conducted. When droplets of an acid solution (9 mL) were dropped from a height of 30 cm onto each absorbent (4 × 4 × 1 cm^3^) for 1 min, the droplet fragments that bounced from the surface of each absorbent were monitored based on the traces left on white paper. Many droplet fragments bounced and were sprinkled around the absorbents when the pad and pristine MFS were used (Figure 7a,b). However, this phenomenon was rarely observed with MFS/IO(3) (Figure 7c). After testing, the white paper was analyzed to count the number of droplet fragments. Many droplet fragments, i.e., 138 and 246, were observed for the pad and pristine MFS, respectively, while only 65 droplet fragments were observed for MFS/IO(3) (Figure 7d–g). These results indicate that the needlelike IO morphology of MFS/IO is an ideal surface morphology that rarely makes droplet fragments when droplets are dropped onto the surface of MFS/IO. To prove this hypothesis, superhydrophilic Cu meshes with smooth and needlelike surfaces were used for droplet fragment tests. The Cu mesh with a smooth surface resulted in many droplet fragments around the mesh, which was also observed with the pad and pristine MFS, while the Cu mesh with a needlelike surface rarely produced fragments (Figure 7h,i). These results further confirmed the abovementioned conclusion. Preventing the formation of droplet fragments as a solution drops onto an absorbent is crucial to avoid secondary contamination from droplet fragments. The abovementioned results indicate that upon collision of a droplet, the droplet exerts a constant force in a certain area on a smooth surface (Figure 7j). This force is not fully absorbed by the smooth surface, causing droplets to bounce and spray, i.e., the principal of action and reaction. However, this force is dispersed by the needlelike structure (Figure 7k). Furthermore, the force can be easily cancelled out because a needlelike morphology uses capillary action to absorb the droplet. After dropping an acid solution on the pad and pristine MFS for 1 min, both adsorbents began to overflow with excess acid solution, and the background papers were slightly wet (Figure 7l). After 5 min, the papers were remarkably wet due to overflow from the absorbents (Figure 7m,n), showing that the absorption capacities of these absorbents are not enough to absorb 9 mL of the acid solution. However, no overflow was observed during the entire span of time for MFS/IO(3) (Figure 7l–n), which suggests that the absorption capacity of MFS/IO(3) for the acid solution is over 9 mL. From the abovementioned results, we concluded that MFS/IO(3) is able to prevent the formation of droplet fragments and solution overflow due to its unique surface morphology and extremely high absorption capacity, respectively.

## 4. Conclusions

MFS/IO(1–5) absorbents with various IO layer thicknesses were synthesized. The MFS/IO absorbents exhibited excellent absorption characteristics toward strong base and acid solutions due to the capillary action between the solution and the needlelike IO structure. The MFS/IO absorbents quickly and naturally absorbed a strong base solution (50%) within 0.5 s and became submerged under the solution without an additional external force during the absorbent drop tests. They also immediately absorbed a strong acid solution (15%) without absorption delay and overflow during solution drop tests due to their fast absorption speeds and sufficient maximum absorption capacities, respectively. These MFS/IO absorbents were demonstrated to be ideal absorbents that generated fewer dust particles than the standard level. MFS/IO(4) in the static mode test generated the lowest concentration (280 piece/L) of dust particles that met the semiclass 1 (ISO 3) level. MFS/IO(3) in the dynamic mode test generated more dust particles (1766–2090 piece/L) than the MFS/IO samples in the static mode tests, but the dust particle concentrations were class 100 level. Furthermore, the MFS/IO absorbents were able to prevent the formation of droplet fragments and solution overflow during the solution drop test owing to their unique surface morphology and extremely high absorption speed/capacity, respectively. We believe that MFS/IO can be used as an ideal absorbent for strong acid and base solutions with extremely high concentrations.

## Supplementary Materials

The following are available online at https://www.mdpi.com/1996-1944/12/20/3389/s1, Figure S1. Weight data of various absorbents. All absorbents had a same dimensional size and volume. The measurements were repeated three times and average values were used; Figure S2. Recyclability test of (a,b) MFS/IO(1–5) and (c,d) MFS/SnO2(1–3) for (a,c) 2nd and (b,d) 3rd use for the absorption of a NaOH solution (50%); Figure S3. SEM images of MFS/IO(1–5) after the 1st absorption of a NaOH solution (50%). (a) MFS/IO(1), (b) MFS/IO(2), (c) MFS/IO(3), (d) MFS/IO(4), and (e) MFS/IO(5); Figure S4. SEM images of MFS/Fe3O4 (a) before and (b) after dropping MFS/Fe3O4 on a NaOH solution (50%); Figure S5. SEM images of MFS/SnO2 (a–c) before and (d–f) after the 1st absorption cycle of a NaOH solution (50%). (a,d) MFS/SnO2(1), (b,e) MFS/SnO2(2), and (c,f) MFS/SnO2(3); Figure S6. Contact angle and absorption time data of a droplet of 50% NaOH solution upon dropping a droplet on various absorbents or substrates. (a1–a5) Superhydrophilic Cu mesh with a smooth surface and (a) its SEM image, (b1–b5) MFS, (c1–c5) absorption pad, (d1–d5) superhydrophilic Cu mesh with a needlelike surface and (d) its SEM image, (e1–e5) MFS/IO(1), and (f1–f5) MFS/IO(5). Each inset shows the WCA of each absorbent or substrate; Figure S7. Drop tests of absorbents on a HCl solution (15%). (a) MFS and (b) absorption pad. Figure S8. Recyclability test of MFS/IO(1–5) for absorption of a HCl solution (15%). (a1–a5) 1st absorption, (b1–b5) 2nd absorption, and (c1–c5) 3rd absorption cycle. (a1,b1,c1) MFS/IO(1), (a2,b2,c2) MFS/IO(2), (a3,b3,c3) MFS/IO(3), (a4,b4,c4) MFS/IO(4), and (a5,b5,c5) MFS/IO(5).

## References

[1] Gu Z., Atherton J.J., Xu Z.P. (2015). Hierarchical Layered Double Hydroxide Nanocomposites: Structure, Synthesis and Applications. Chem. Commun..

[2] Wang H., Hao Q., Yang X., Lu L., Wang X. (2010). A Nanostructured Graphene/Polyaniline Hybrid Material for Supercapacitors. Nanoscale.

[3] Kurt C., Bittner J. (2003). Sodium Hydroxide in Ullmann’s Encyclopedia of Industrial Chemistry.

[4] Ali M.K.A., Xianjun H., Abdelkareem M.A.A., Gulzar M., Elsheikh A.H. (2018). Novel Approach of the Graphene Nanolubricant for Energy Saving via Anti-Friction/Wear in Automobile Engines. Tribol. Int..

[5] Lewinsky A.A. (2007). Hazardous Materials Wastewater.

[6] Blackman W.C. (2016). Basic Hazardous Waste Management.

[7] Swannell R.P., Lee K., McDonagh M. (1996). Field Evaluations of Marine Oil Spill Bioremediation. Microbiol. Rev..

[8] Basak S., Nanda J., Banerjee A. (2012). A New Aromatic Amino Acid Based Organogel for Oil Spill Recovery. J. Mater. Chem..

[9] Vidyasagar A., Handore K., Sureshan K.M. (2011). Soft Optical Devices from Self-Healing Gels Formed by Oil and Sugar-Based Organogelators. Angew. Chem..

[10] Mukherjee S., Mukhopadhyay B. (2012). Phase Selective Carbohydrate Gelator. RSC Adv..

[11] Xu Z., Zhao Y., Wang H., Wang X., Lin T. (2015). A Superamphiphobic Coating with an Ammonia-Triggered Transition to Superhydrophilic and Superoleophobic for Oil–Water Separation. Angew. Chem..

[12] Kaang B.K., Han N., Jang W., Koo H.Y., Lee Y.B., Choi W.S. (2018). Crossover Magnetic Amphiprotic Catalysts for Oil/Water Separation, the Purification of Aqueous and Non-Aqueous Pollutants, and Organic Synthesis. Chem. Eng. J..

[13] Wang Y., Shi Y., Pan L., Yang M., Peng L., Zong S., Shi Y., Yu G. (2014). Multifunctional Superhydrophobic Surfaces Templated from Innately Microstructured Hydrogel Matrix. Nano Lett..

[14] Zhang W., Shi Z., Zhang F., Liu X., Jin J., Jiang L. (2013). Superhydrophobic and Superoleophilic PVDF Membranes for Effective Separation of Water-in-Oil Emulsions with High Flux. Adv. Mater..

[15] Han N., Lim Y.T., Jang W., Koo H.Y., Choi W.S. (2016). Polydopamine-Mediated All-in-One Device with Superhydrophilicity and Superhydrophobicity for One-Step Oil/Water Separation and Pollutant Purification. Polymer.

[16] Pan Q., Wang M., Wang H. (2008). Separating Small Amount of Water and Hydrophobic Solvents by Novel Superhydrophobic Copper Meshes. Appl. Surf. Sci..

[17] Wang C., Yao T., Wu J., Ma C., Fan Z., Wang Z., Cheng Y., Lin Q., Yang B. (2009). Facile Approach in Fabricating Superhydrophobic and Superoleophilic Surface for Water and Oil Mixture Separation. ACS Appl. Mater. Interfaces.

[18] Crick C.R., Gibbins J.A., Parkin I.P. (2013). Superhydrophobic Polymer-Coated Copper-Mesh; Membranes for Highly Efficient Oil–Water Separation. J. Mater. Chem. A.

[19] Boakye-Ansah S., Lim Y.T., Lee H.-J., Choi W.S. (2016). Structure-Controllable Superhydrophobic Cu Meshes for Effective Separation of Oils with Different Viscosities and Aqueous Pollutant Purification. RSC Adv..

[20] Li L., Hu T., Sun H., Zhang J., Wang A. (2017). Pressure-Sensitive and Conductive Carbon Aerogels from Poplars Catkins for Selective Oil Absorption and Oil/Water Separation. ACS Appl. Mater. Interfaces.

[21] Li L., Li B., Sun H., Zhang J. (2017). Compressible and Conductive Carbon Aerogels from Waste Paper with Exceptional Performance for Oil/Water Separation. J. Mater. Chem. A.

[22] Islam M.S., Choi W.S., Kim S.H., Han O.H., Lee H.-J. (2015). Inorganic Micelles (Hydrophilic Core@Amphiprotic Shell) for Multiple Applications. Adv. Funct. Mater..

[23] Zhang J., Li B., Li L., Wang A. (2016). Ultralight, Compressible and Multifunctional Carbon Aerogels Based on Natural Tubular Cellulose. J. Mater. Chem. A.

[24] Lee Y.S., Kaang B.K., Han N., Lee H.-J., Choi W.S. (2018). An Anti-Overturn Janus Sponge with Excellent Floating Stability for Simultaneous Pollutant Remediation and Oil/Water Separation. J. Mater. Chem. A.

[25] Zhang Y.Q., Yang X.B., Wang Z.X., Long J., Shao L. (2017). Designing Multifunctional 3D Magnetic Foam for Effective Insoluble Oil Separation and Rapid Selective Dye Removal for Use in Wastewater Remediation. J. Mater. Chem. A.

[26] Zhu H., Chen D., Li N., Xu Q., Li H., He J., Lu J. (2017). Dual-Layer Copper Mesh for Integrated Oil-Water Separation and Water Purification. Appl. Catal. B.

[27] Bao C., Bi S., Zhang H., Zhao J., Wang P., Yue C., Yang J. (2016). Graphene Oxide Beads for Fast Clean-Up of Hazardous Chemicals. J. Mater. Chem. A.

[28] Doering R., Nishi Y. (2007). Handbook of Semiconductor Manufacturing Technology.

[29] Chen W., Lin J., Hu G., Han X., Liu M., Yang Y., Wu Z., Liu Y., Zhang B. (2015). GaN Nanowire Fabricated by Selective Wet-Etching of GaN Micro Truncated-Pyramid. J. Cryst. Growth.

[30] Jang S., Jung S., Beers K., Yang J., Ren F., Kuramata A., Pearton S.J., Baik K.H. (2018). A Comparative Study of Wet Etching and Contacts on (201) and (010) Oriented β-Ga_2_O_3_. J. Alloys Compd..

[31] Samsung Electronics (2017). Standards for Control of Substances Used in Products, Revision 19, SEC Registration NO. 0QA-2049.

[32] Kaang B.K., Han N., Lee H.-J., Choi W.S. (2018). Polyelectrolyte brush-grafted polydopamine-based catalysts with enhanced catalytic activity and stability. ACS Appl. Mater. Interfaces.

[33] Bae J.Y., Lee H.-J., Choi W.S. (2016). Cube sugar-like sponge/polymer brush composites for portable anduser-friendly heavy metal ion adsorbents. J. Hazard. Mater..

[34] Moya S., Azzaroni O., Farhan T., Osborne V.L., Huck W.T.S. (2005). Locking and Unlocking of Polyelectrolyte Brushes: Toward the Fabrication of Chemically Controlled Nanoactuators. Angew. Chem. Int. Ed..

[35] Han N., Lee Y.S., Kaang B.K., Jang W., Koo H.Y., Choi W.S. (2019). A lottery draw machine-inspired movable air filter with high removal efficiency and low pressure drop at high flow rate. J. Mater. Chem A.

[36] Lim Y.T., Han N., Jang W., Jung W., Oh M., Han S.W., Koo H.Y., Choi W.S. (2017). Surface Design of Separators for Oil/Water Separation with High Separation Capacity and Mechanical Stability. Langmuir.

[37] Hu J.-S., Zhong L.-S., Song W.-G., Wan L.-J. (2008). Synthesis of Hierarchically Structured Metal Oxides and their Application in Heavy Metal Ion Removal. Adv. Mater..

[38] Zhong L.-S., Hu J.-S., Liang H.-P., Cao A.-M., Song W.-G., Wan L.-J. (2006). Self-Assembled 3D Flowerlike Iron Oxide Nanostructures and Their Application in Water Treatment. Adv. Mater..

