# Supplementary material for: An Active Absorbent for Cleanup of High-Concentration Strong Acid and Base Solutions
Source: Materials (Basel). 2019 Oct 17;12(20):3389. doi: 10.3390/ma12203389 (PMC6829578; doi:10.3390/ma12203389)
Supplement: Supplementary file 1 [file materials-12-03389-s001.pdf]

materials

Supporting Information

An Active Absorbent for Cleanup of High-Concentration Strong Acid and Base Solutions

Nara Han [1], Sol Park [1], Byung Kwon Kaang [1], Wooree Jang [2], Hye Young Koo [2,*] and Won San Choi [1,*]

1 Department of Chemical and Biological Engineering, Hanbat National University, 125 Dongseodaero, Yuseong-gu, Daejeon 305-719, Korea; xoc123@naver.com (N.H.); thf6363@naver.com (S.P.); rkdqudrnjs3@naver.com (B.K.K.)
2 Functional Composite Materials Research Center, Korea Institute of Science and Technology (KIST) Jeonbuk Institute of Advanced Composite Materials, 92 Chudong-ro, Bongdong-eup, Wanju-gun, Jeollabuk-do55324, Korea; 215015@kist.re.kr
* Correspondence: koohy@kist.re.kr (H.Y.K.); choiws@hanbat.ac.kr (W.S.C.)

Experimental section

Preparation of MFS/Fe_3O_4

$FeCl_36H_2O$ (0.9 g) and CH_3COONa (2.4 g) were dissolved in ethylene glycol (30 mL) under vigorous stirring for 5 min at room temperature. The resulting solution was transferred to a Teflon-lined autoclave bomb and heated in an oven at 200 °C for 8 h. The final product was washed 3 times with ethanol and DI water. A mixture containing EtOH (100 mL) and MNPs (200 mg) was stirred for 1 min. Then, an MFS (2 × 2 × 2 cm^3) was added to the mixture, followed by ultrasonication for 10 min. After 10 min, the resulting sponge was dried in an oven at 50 °C without washing. The resulting MFS/MNP was washed 3 times with DI water and dried in an oven at 50 °C.

Preparation of MFS/Au or Ag

Gold(III) chloride trihydrate or silver(I) nitrate (0.066 g) was dissolved in EtOH (17 mL) under vigorous stirring for 5 min at room temperature. Then, an MFS (2 × 2 × 2 cm^3) was added to the resulting solution, followed by stirring for 4 h. After 4 h, the resulting sponge was washed 3 times with DI water. The resulting sponge was dipped in $NaBH_4$ (10 mM) solution for 30 min, washed 3 times with DI water, and dried in an oven at 50 °C.

Preparation of MFS/TiO_2

Titanium tertbutoxide (7.1 mL) was dissolved in EtOH (50 mL) under vigorous stirring for 1 min at room temperature. Then, 1 mL of DI water was added to the solution, which was stirred for 30 min, and 0.5 mL of HCl solution (pH=3) was added to the resulting solution. An MFS (2 × 2 × 2 cm^3) was added to the resulting solution, followed by stirring for 4 h. After 4 h, the resulting sponge was washed 3 times with DI water and dried in an oven at 50 °C.

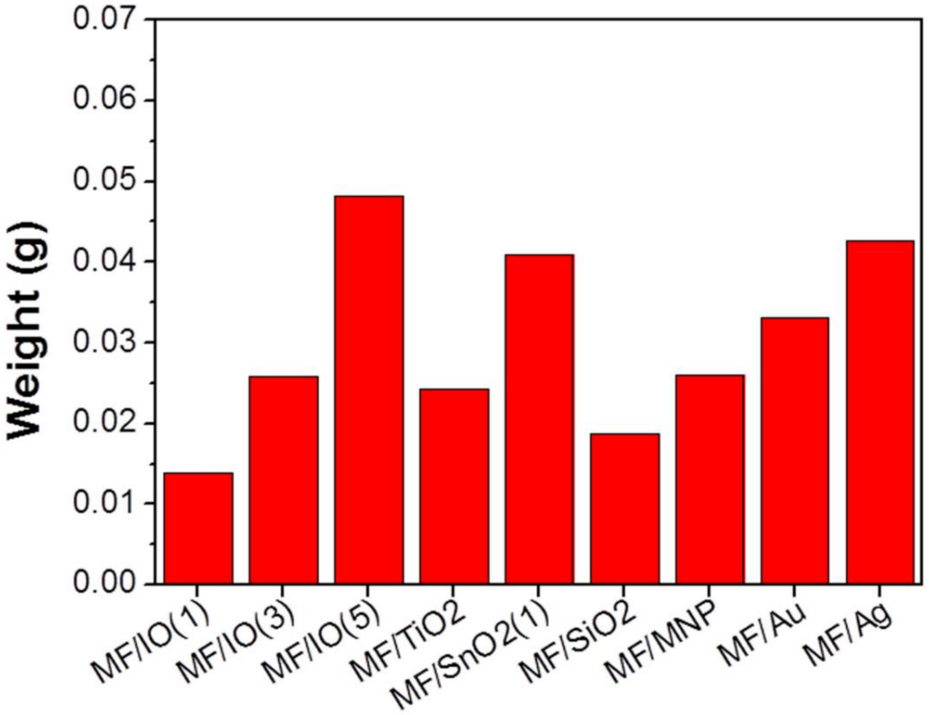

Figure S1. Weight data of various absorbents. All absorbents had a same dimensional size and volume. The measurements were repeated three times and average values were used.

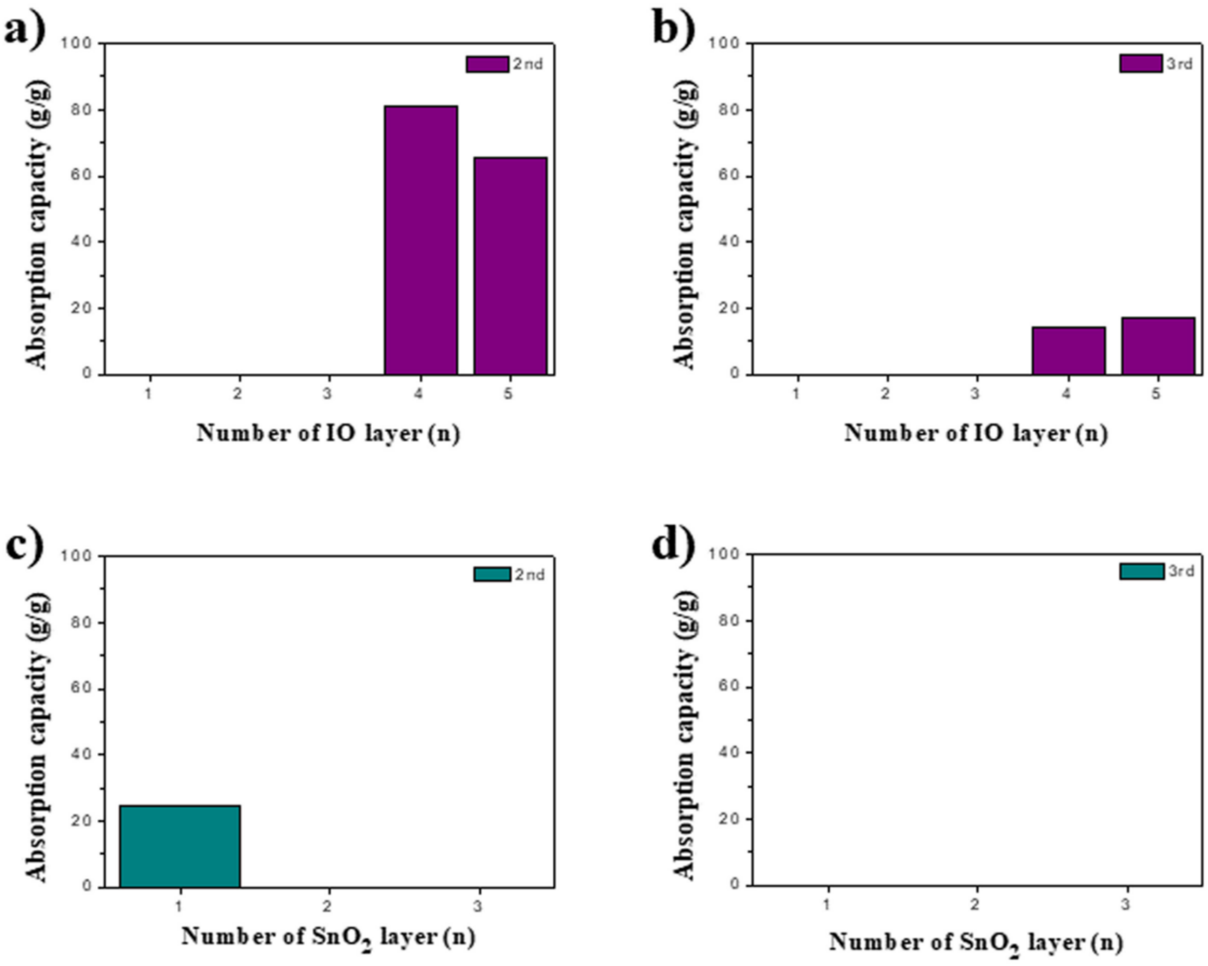

Figure S2. Recyclability test of (**a**,**b**) MFS/IO(1–5) and (**c**,**d**) MFS/SnO_2(1–3) for (**a**,**c**) 2nd and (**b**,**d**) 3rd use for the absorption of a NaOH solution (50%).

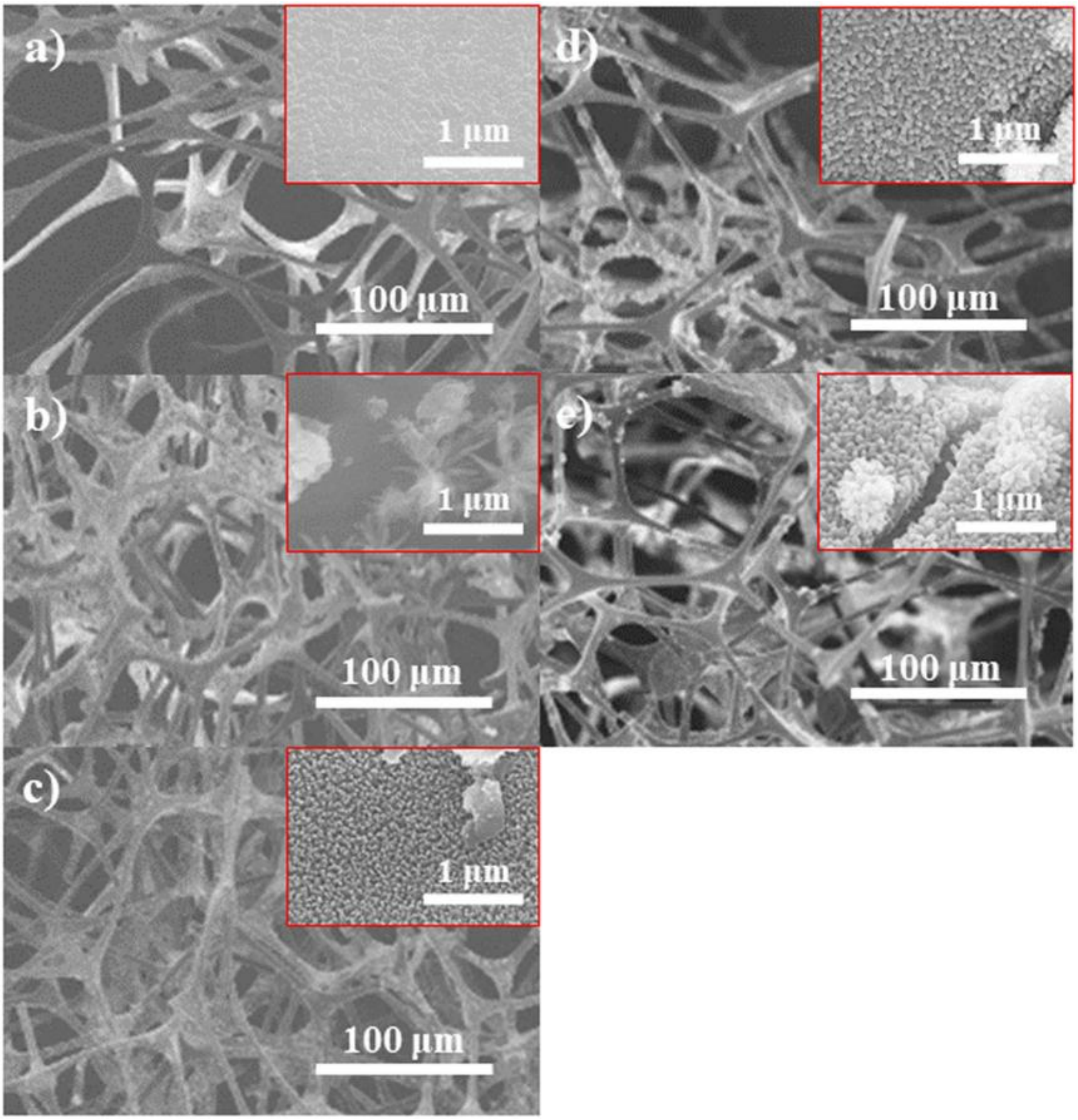

Figure S3. SEM images of MFS/IO(1−5) after the 1st absorption of a NaOH solution (50%). (**a**) MFS/IO(1), (**b**) MFS/IO(2), (**c**) MFS/IO(3), (**d**) MFS/IO(4), and (**e**) MFS/IO(5).

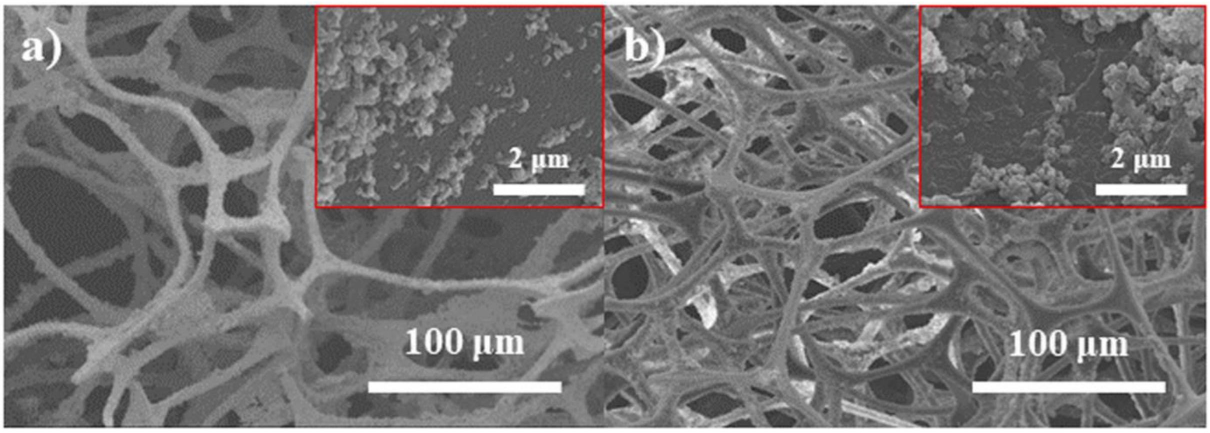

Figure S4. SEM images of MFS/Fe_3O_4 (**a**) before and (**b**) after dropping MFS/Fe_3O_4 on a NaOH solution (50%).

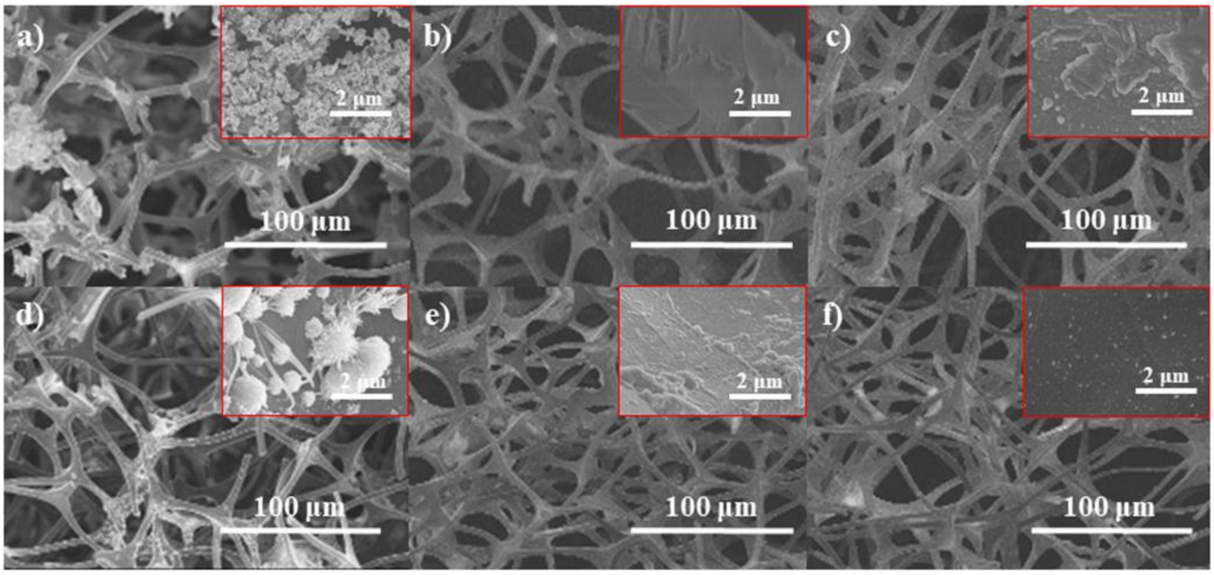

Figure S5. SEM images of MFS/SnO_2 (**a**–**c**) before and (**d**–**f**) after the 1st absorption cycle of a NaOH solution (50%). (**a**,**d**) MFS/SnO_2(1), (**b**,**e**) MFS/SnO_2(2), and (**c**,**f**) MFS/SnO_2(3).

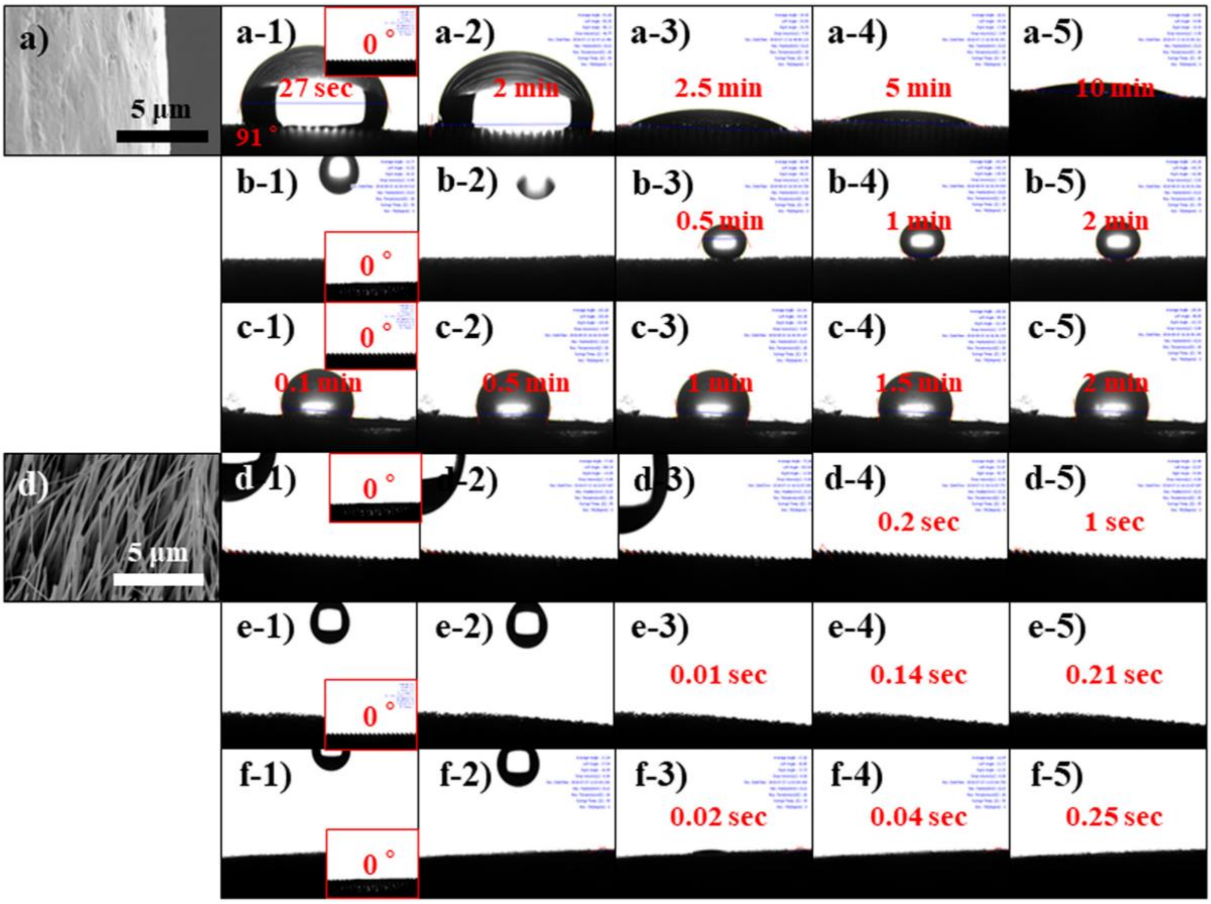

Figure S6. Contact angle and absorption time data of a droplet of 50% NaOH solution upon dropping a droplet on various absorbents or substrates. (**a1**–**a5**) Superhydrophilic Cu mesh with a smooth surface and (**a**) its SEM image, (**b1**–**b5**) MFS, (**c1**–**c5**) absorption pad, (**d1**–**d5**) superhydrophilic Cu mesh with a needlelike surface and (**d**) its SEM image, (**e1**–**e5**) MFS/IO(1), and (**f1**–**f5**) MFS/IO(5). Each inset shows the WCA of each absorbent or substrate.

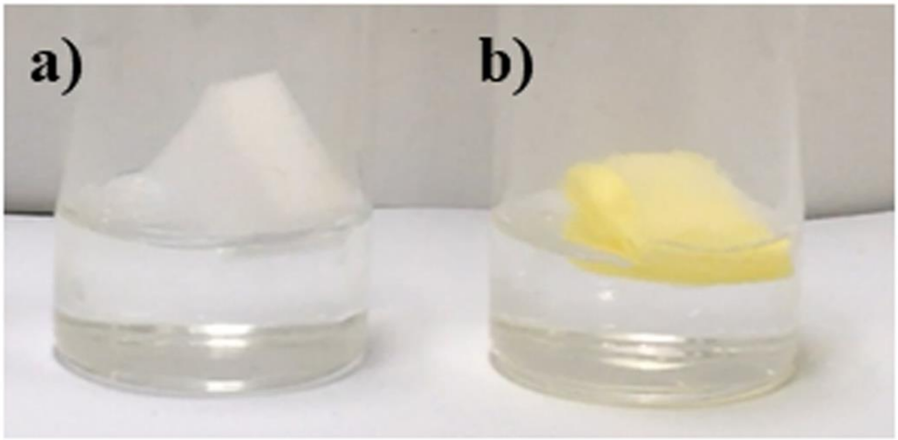

Figure S7. Drop tests of absorbents on a HCl solution (15%). (**a**) MFS and (**b**) absorption pad.

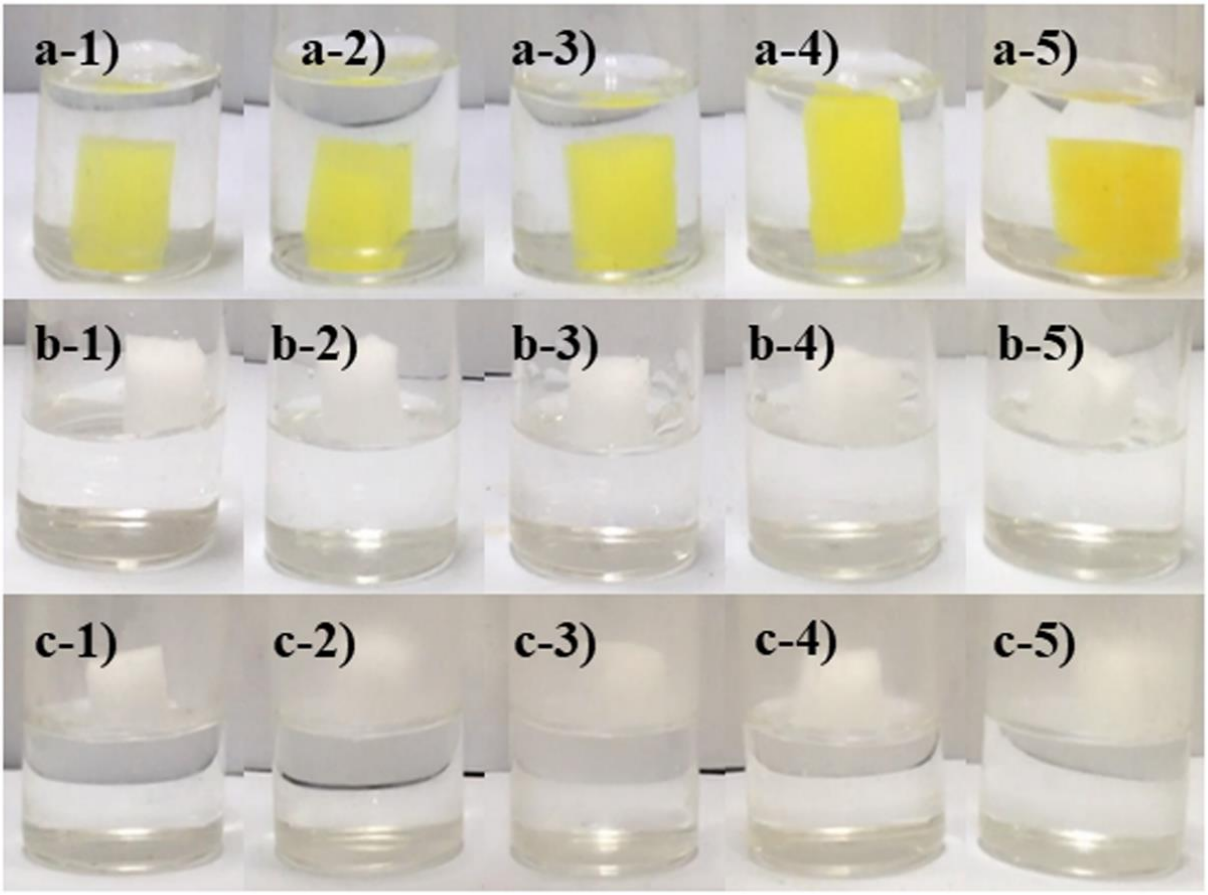

Figure S8. Recyclability test of MFS/IO(1−5) for absorption of a HCl solution (15%). (**a1**−**a5**) 1st absorption, (**b1**–**b5**) 2nd absorption, and (**c1**–**c5**) 3rd absorption cycle. (**a1**,**b1**,**c1**) MFS/IO(1), (**a2**,**b2**,**c2**) MFS/IO(2), (**a3**,**b3**,**c3**) MFS/IO(3), (**a4**,**b4**,**c4**) MFS/IO(4), and (**a5**,**b5**,**c5**) MFS/IO(5).
